# Effect of metabotropic glutamate receptor 3 genotype on N-acetylaspartate levels and neurocognition in non-smoking, active alcoholics

**DOI:** 10.1186/1744-9081-8-42

**Published:** 2012-08-21

**Authors:** Yan Xia, Dongying Ma, Jian Hu, Chunling Tang, Zheng Wu, Lei Liu, Feng Xin

**Affiliations:** 1 Mental Health Institute, Mental Health Centre, 1st Affiliated Hospital of Harbin Medical University, 23 Youzheng Street, Harbin, Heilongjiang Province, 150001, PR China; 2 Department of Neurosurgery, 2nd Affiliated Hospital of Harbin Medical University, 246 Xuefu Road, Harbin, Heilongjiang Province, 150086, PR China

**Keywords:** Alcohol dependence, Metabotropic glutamate receptor 3, Single nucleotide polymorphism, N-acetylaspartate, Executive function

## Abstract

**Background:**

We studied the effects of single nucleotide polymorphisms (SNPs) in the metabotropic glutamate receptor 3 (GRM3) gene on brain N-acetylaspartate (NAA) concentrations and executive function (EF) skills in non-smoking, active alcoholics, and evaluated associations between these variables.

**Methods:**

SNPs (rs6465084, rs1468412, and rs2299225) in GRM3 were genotyped in 49 male, non-smoking, alcohol-dependent patients and 45 healthy control subjects using ligase detection reactions. NAA/creatine (Cr) ratios in left prefrontal gray matter (GM) and white matter (WM), left parietal GM, left parietal WM, and cerebellar vermis regions were measured by Proton ^1^ H Magnetic resonance spectroscopy (MRS). EF was measured by the Wisconsin Card Sorting Test (WCST).

**Results:**

Compared to controls, alcoholics had lower NAA/Cr ratios in prefrontal GM and WM regions and performed more poorly on all EF tests (*P* < 0.001). Alcoholics with the A/A genotype for SNP rs6465084 had lower NAA/Cr ratios in prefrontal GM and WM regions and had poorer EF skills than alcoholics who were G-carriers for this SNP (*P* < 0.01). Non-alcoholics with the A/A genotype for rs6465084 also had lower NAA/Cr levels in prefrontal GM and made more random errors in the WCST than G-carriers (*P* < 0.01). The A/A genotype group for SNP rs6465084 was significantly different from the G carriers for the variables of NAA/Cr ratios and WCST scores in both alcoholics and controls (*P* < 0.05). Alcoholics who were T-carriers for rs1468412 had lower NAA/Cr ratios in prefrontal GM and showed poorer EF skills (*P* < 0.05). No effects of rs2299225 genotype on NAA/Cr or executive skills were observed. NAA/Cr in left prefrontal regions correlated with certain parameters of EF testing in both alcoholics and controls (*P* < 0.05), but the significance of this correlation among alcoholics disappeared after adjustment for the effects of genotype.

**Conclusions:**

Our results provide evidence that glutamate system dysfunction may play a role in the prefrontal functional abnormalities seen in alcohol dependence. It is possible that certain GRM3 SNP genotypes (the A/A genotype of rs6465084 and the T allele of rs1468412) may further lower NAA/Cr levels and EF skills in addition to the effect of alcohol.

## Background

Alcohol dependence and abuse are among the most costly health problems in the world from both social and economic points of view, resulting from interplay among polygenic, sociocultural, and environmental factors. The long-term effects of chronic alcohol abuse on brain damage and cognition have been well documented [1-3]. Genetic factors may play a role in the vulnerability to and severity of alcohol-induced brain damage [4,5].

Alcohol dependence is partly due to dysregulation of glutamate transmission in the brain [6,7]. Glutamate is the major excitatory neurotransmitter in the central nervous system and mediates its actions via activation of both ionotropic and metabotropic receptor families. Metabotropic glutamate receptors (mGlus) have received much attention in the neuropsychiatric community over the past decade, and have become targets of drug developers searching for medicines to treat schizophrenia, depression, anxiety, drug addiction, and dementia [8,9]. mGlus are divided into three groups, mGluI-mGluIII, based on signal transduction pathways and sequence homology [10]. The metabotropic glutamate receptor 3 (GRM3), a member of the group II family of mGlus, is coupled to second message pathways via GTP-binding proteins and is involved in presynaptic depression by decreasing the evoked release of glutamate [11].

The GRM3 gene has been mapped to 7q21.1–q21.2, spanning 220.1 kb [12], and is a candidate gene for susceptibility to schizophrenia. The single nucleotide polymorphism (SNP) rs6465084 in intron 2 has been associated with reduced prefrontal cortical levels of N-acetylaspartate/ Creatine (NAA/Cr) and verbal fluency in schizophrenic and healthy subjects [13,14]. Two SNPs, rs1468412 and rs2299225 in intron 3, were reported to be associated with schizophrenia in Japanese and Chinese populations, respectively [11,15].

Several animal studies have looked at the influence of GRM3 activation on alcohol dependence and addiction to other drugs [16-21]. In one study, the mGlu2/3 receptor agonist LY379268 attenuated alcohol self-administration and cue-induced reinstatement [16]. Another study demonstrated that the same GRM2/3 agonist (LY379268) was effective at reducing ethanol self-administration and stress-induced reinstatement following dependence-inducing ethanol exposure [21]. Furthermore, GRM3 mRNA levels were found to be down-regulated in rat strains genetically selected for alcohol self-administration preference [19]. These results suggest that GRM2/3 receptors may be good targets for treatment of for alcohol addiction.

Magnetic resonance spectroscopy (MRS) is the neuroimaging method of choice for noninvasive monitoring of *in vivo* brain chemistry, and provides a unique opportunity to gain insight into the biochemical pathology of neurological and psychiatric disorders. MRS enables measurement of aspects of alcohol-induced brain damage that may accompany or precede alcohol-induced morphological changes [22-26]. The brain metabolite concentrations measured by MRS include N-acetylaspartate (NAA), choline-containing compounds (Cho), creatine and phosphocreatine (Cr), myo-inositol (mI), and lactate. NAA is an amino acid derivative that is found in high concentrations in axons and dendrites of neurons, particularly in pyramidal neurons [27], and NAA levels are thought to reflect neuronal viability [28]. Decreased NAA levels usually suggest neuronal loss or atrophied dendrites and/or axons [29]. Cr is involved in the bioenergetics of neuronal and glial tissue. Levels of this metabolite generally do not vary with pathological status, making it useful as an internal reference to other brain metabolites [30-34]. Because smoking has been shown to influence NAA/Cr levels regardless of alcohol consumption [23-25,35], we ascertained study participants who were non-smokers to avoid any additional effect of smoking on this investigation. Several studies have suggested that chronic cigarette smoking adversely affects both brain neurobiology and neurocognition in alcohol use disorders. MRS studies comparing smoking and non-smoking recovering alcoholics found lower NAA concentrations in the frontal [23-25] and medial temporal lobes [35] of the smokers. Examining alcoholics as a homogeneous group, without consideration of smoking status, may obscure the real effect of alcohol on neurocognitive injuries.

We hypothesized that chronic alcohol consumption would lead to a measurable reduction in MRS-measured NAA/Cr levels and poor performance on executive function (EF) tests. Furthermore, we speculated that in addition to the direct effect of alcohol, certain SNPs (rs6465084, rs1468412, and rs2299225) in the GRM3 gene may contribute to lower NAA/Cr levels in one or more brain regions and to lower EF skills through modulation of synaptic glutamate. The goal of this study was to investigate the effects of GRM3 SNP genotypes on brain NAA concentrations and EF skills in alcoholics, and evaluated relationships between these parameters.

## Methods

### Participants

Forty-nine male, non-smoking, heavy drinkers were recruited from the Mental Health Centre of 1^st^ Hospital of Harbin Medical University, China. All patients met the DSM-IV criteria for alcohol dependence and consumed more than 70 g of pure ethanol daily for at least 3 years without smoking and were not abstaining from alcohol at enrollment. Forty-five male, non-smoking control subjects were recruited from the same hospital. Alcohol consumption by the control subjects, if any, was consistent with that of a mild social drinker, with a mean of 4 g of pure ethanol per day and a maximum of 12 g per day (one subject). Control subjects were carefully screened for any past or present alcohol- or drug-related problems. All subjects were Han Chinese in origin between the ages of 18 and 60 y at the time of enrollment. Participants were excluded from the study for any concomitant psychiatric disorder, antisocial personality disorder, organic liver failure, and medical or neurological illness or trauma that would affect the central nervous system. This study was approved by the local Ethics Committee, and all subjects provided written informed consent. All subjects, particularly the alcohol-dependent group, were operating at their full cognitive capacities at the time they gave their informed consent.

Alcohol consumption questionnaires were collected from each study participant. Data collected included age of onset of alcohol dependence, duration of alcohol dependence, estimated daily alcohol intake, estimated number of drinks in the prior month, estimated drinks per day and per month in the past year, and lifetime estimated alcohol intake. Pure alcohol intake was computed in drink/day (one drink was defined as 12 oz of beer, 5 oz of wine, or 1.5 oz of liquor, corresponding to approximately 13.6 g of pure alcohol).

### Genotyping

The three SNPs studied in the GRM3 gene, rs6465084(A/G), rs1468412(A/T), and rs2299225(G/T) (http://www.ncbi.nlm.nih.gov/SNP/), reside within introns. Genomic DNA was isolated from whole blood using the AxyPrep-96 genomic DNA kit (Axygen Biotechnology Company, China) according to the manufacturer's instructions. Genotyping was done using ligase detection reactions (LDR). All three target DNA sequences were amplified in a multiplex PCR reaction. The PCR reaction was carried out in a final volume of 20 μl containing 1X PCR buffer, 3 mM Mg^2+^, 2 mM dNTP, 1U/μl Taq DNA ligase (Qiagen Company, Germany), 1X Q-solution, 5 pM primer [see Additional file 1], 1 pmol of each common probe [see Additional file 2], and 1 μl genomic DNA. The reaction conditions were 95°C for 15 m, 35 cycles of 94°C for 30 s, 56°C for 90 s, and 72°C for 1 m, followed by a final extension at 72°C for 7 m. The ligation reaction was carried out in a final volume of 10 μl containing 1X buffer, 1 μl Probe Mix, 0.05 μl ligase, 1 μl Multi-PCR product, and 1 pmol of each discriminating oligo. The LDR reaction conditions were 95°C for 2 m, and 30 cycles of 94°C for 30 s and 50°C for 2 m. The fluorescent products of LDR were differentiated using the ABI sequencer 500.

### Magnetic resonance spectroscopic imaging acquisition and processing

**Figure 1:**
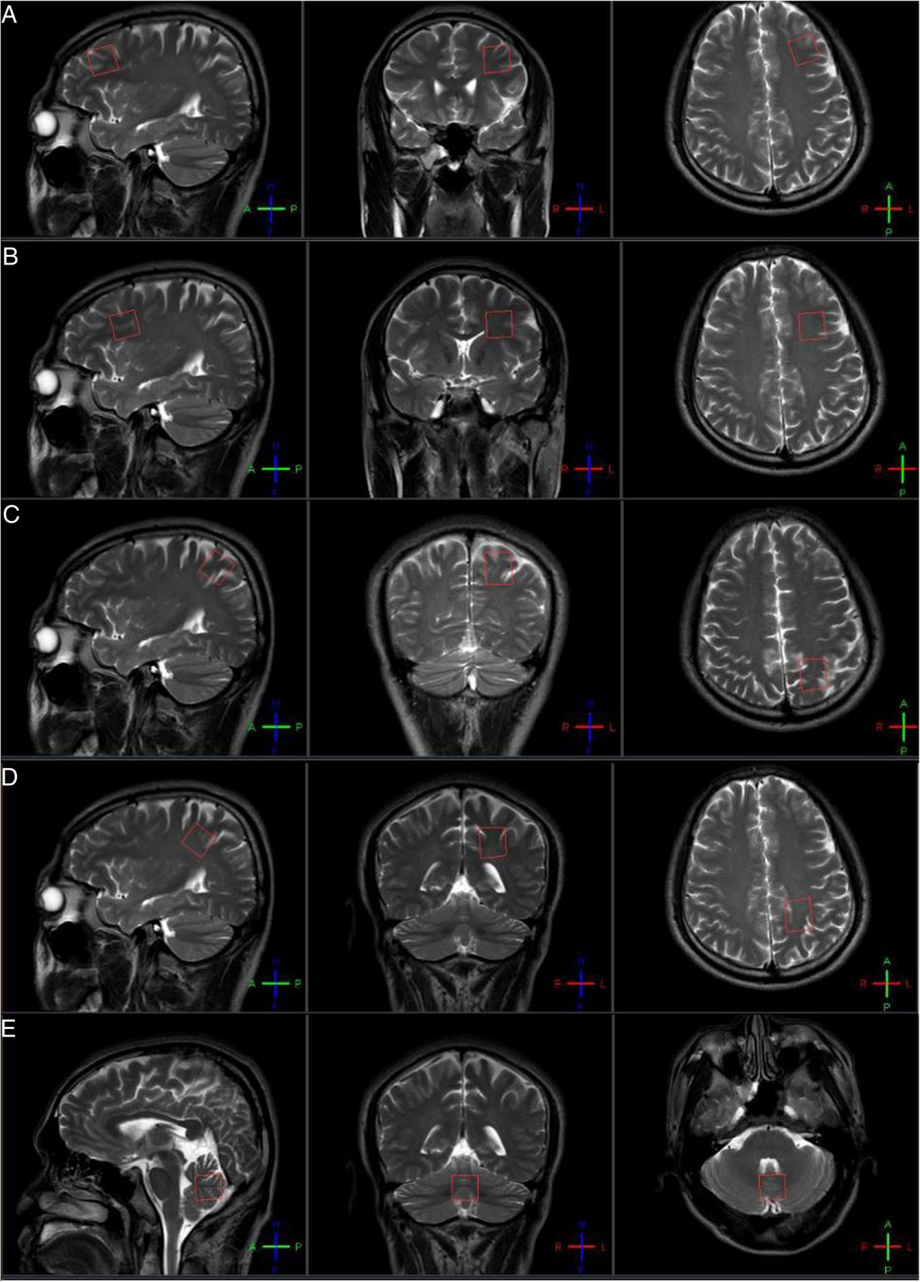
**Regions of interest (RoIs).** RoIs in this study were the left prefrontal GM (**A**), left prefrontal WM (**B**), left parietal GM (**C**), left parietal WM (**D**), and cerebellar vermis (**E**).

Proton magnetic resonance spectroscopic imaging (MRSI) was performed with a 3.0 T Philips Vision system (Philips Company, Netherlands), as described by Meyerhoff et al. [22]. MRI was followed by automated head shimming and a multislice 1 H MRSI sequence with TR, TI, and TE of 1800, 165, and 25 ms, respectively, 15° flip angle, circular *k*-space sampling, and three 15 mm thick slices, with a slice gap of at least 6 mm, angulated parallel to the double spin-echo slices. The first MRSI plane was positioned through the cerebellum and pons using two anatomical landmarks for reproducible slice positioning on the sagittal view. The junction of the pons and midbrain and the corner of the fourth ventricle and vermis were used to determine the angulation and position of the oblique transverse slice through the cerebellum and pons. The second slice was positioned superior to the lateral ventricles including frontal lobe gray and white matter and the anterior cingulate gyrus. Regions of interest (RoIs, 20 mm × 20 mm × 20 mm) were drawn on the left prefrontal gray matter (GM), left prefrontal white matter (WM), left parietal GM, left parietal WM, and the cerebellar vermis (five brain regions most susceptible to alcohol consumption) with an atlas-based method (Figure 1). The total acquisition time was approximately 45 minutes for both MRI and MRSI.

**Figure 2:**
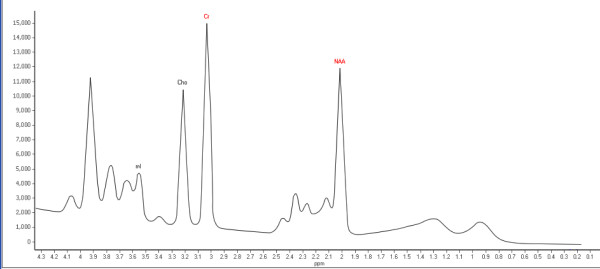
**Representative spectrum showing NAA and Cr measurements.** Peak integral values were determined by a fit curve algorithm at 2.0 ppm for NAA and 3.0 ppm for Cr.

Metabolite signals of NAA and Cr were calculated from the MRSI data using an automated spectral fitting program (Philips Company, the Netherlands). Metabolite signal intensities were reported as the area under the peaks and used to calculate NAA/Cr ratio. Cr values were used as a reference because the total amount of Cr appears to be constant under different metabolic conditions [31]. Moreover, total Cr resonance is generally not expected to be affected by alcohol [32-34]. A representative spectrum and its fit after subtraction of the fitted baseline are depicted in Figure 2.

### Neurocognitive assessment

Wisconsin Card Sorting Test (WCST) was administered within 1 day of the magnetic resonance study. The test (~20 min in duration) is a commonly used instrument to measure frontal EF such as concept formation, setshifting, and flexibility. Using a computerized 48-card version of the WCST, the subjects were required to match response cards to 4 stimulus cards along 1 of 3 dimensions (color, form, or number) on the basis of verbal feedback (correct or incorrect). The participants were not given any information about the dimensions. After a certain number of correct answers, the sorting rule was changed without warning. The output parameters of the WCST included number of correct responses, perseverative errors, random errors, and categories completed.

### Statistical analyses

The effects of GRM3 genotype on cognitive abilities and NAA/Cr ratio measures were assessed using the combination of unpaired *t* tests and multivariate analysis of variance (MANOVA). Many genotypes were examined in this data set, but Bonferroni correction for all combinations seems overly stringent [13]. The results of unpaired *t* tests were corrected for multiple tests using MANOVA. Potential confounding factors (demographic and clinical variables) were also examined but were not significant. The variables were all normally distributed, thus the samples were evaluated using Pearson correlation coefficients to assess the association between NAA/Cr levels and changes in EF skills, with or without the adjustment of variables by genotypes.

## Results

### Participant characteristics

Demographics and estimated alcohol consumption in alcoholic and control group are shown in Table 1. Genotype frequencies for the three SNPs in GRM3 are shown in Table 2. The age, education, and genotype frequencies were not significantly different between alcoholics and controls. Subjects in different genotype groups did not differ significantly by age, education, or alcohol consumption.

**Table 1:** Demographics and estimated alcohol consumption in alcoholic and control group

**Variable**	**Alcoholic group**	**Control group**
Age	46.0 ± 6.5	46.3 ± 5.8
Education (years)	11.7 ± 3.5	11.9 ± 3.0
Onset age of alcohol dependence	28.5 ± 5.2	NA
Duration of alcohol dependence (years)	17.3 ± 6.2	NA
Estimated average drinks ^** *a* **^ per day in prior month	11 ± 5	0.4 ± 0.2
Estimated drinks in prior month	324 ± 143	11 ± 7
Estimated drinks per day last year	11 ± 5	0.4 ± 0.2
Estimated drinks per month last year	358 ± 153	11 ±7
Estimated lifetime alcohol drinks	85430 ± 57290	3432 ± 1399

NA: Not applicable.

^** *a* **^ One drink is equivalent to 12 oz beer, 5 oz wine, or 1.5 oz liquor containing approximately 13.6 g of ethanol.

**Table 2:** Genotype frequencies of three SNPs in the GRM3 gene in alcoholics and controls

**SNP**	**Genotype, number and frequency**		
**Alcoholic group**			
rs6465084	A/A	A/G	G/G
	31 (63.3%)	17 (34.7%)	1 (2.0%)
rs1468412	A/A	A/T	T/T
	30 (61.2%)	17 (34.7%)	2 (4.1%)
rs2299225	T/T	G/T	G/G
	37 (75.5%)	12 (24.5%)	0
**Control group**			
rs6465084	A/A	A/G	G/G
	29 (64.4%)	16 (35.6%)	0
rs1468412	A/A	A/T	T/T
	27 (60.0%)	17 (37.8%)	1 (2.2%)
rs2299225	T/T	G/T	G/G
	30 (66.7%)	15 (33.3%)	0

### Brain metabolite levels and neurocognition test results for alcohol-dependent and control subjects

The combination of unpaired *t* tests and MANOVA were used in this analysis. NAA/Cr ratio measurements in each RoI and WCST scores for alcoholics and controls are given in Table 3. The alcohol-dependent group differed from the controls (*F* = 17.18, v1 = 9, v2 = 82, *P* < 0.001) for the variables which included all NAA/Cr levels and WCST scores in the MANOVA. The alcohol-dependent group was also different from the controls (*F* = 8.12,v1 = 5, v2 = 86, *P* < 0.001) only for the variables of NAA/Cr levels. The alcohol-dependent group (*n* = 49) had lower NAA/Cr levels in left prefrontal GM (*t* = −2.59, *df* = 92, *P* = 0.011) and in the prefrontal WM (*t* = −6.03, *df* = 80, *P* < 0.001) compared to the controls (*n* = 45) using unpaired *t* tests. The NAA/Cr ratios in the other brain RoIs were not significantly different between the groups. The alcohol-dependent group differed from the controls (*F* = 37.74, v1 = 4, v2 = 89, *P* < 0.001) for the variables of WCST scores in the MANOVA. The alcohol-dependent group performed more poorly than control subjects in the number of correct responses (*t* = −11.58, *df* = 82, *P* < 0.001), perseverative errors (*t* = 8.87, *df* = 92, *P* < 0.001), random errors (*t* = 4.70, *df* = 72, *P* < 0.001), and categories completed (*t* = −11.52, *df* = 65, *P* < 0.001, unpaired *t* tests).

**Table 3:** NAA/Cr ratio measurements and WCST scores in alcoholics and controls

**Parameter**	**Alcoholic group ( *n* ** **= 49)**	**Control group ( *n* ** **= 45)**	** *t* **	** *df* **	** *P* **
Left Prefrontal GM	1.59 ± 0.13	1.66 ± 0.12	−2.59	92	0.011^*a*^
Left Prefrontal WM	1.58 ± 0.12	1.70 ± 0.07	−6.03	80	<0.001^*b*^
Left Parietal GM	1.53 ± 0.09	1.55 ± 0.07	−1.24	91	0.220
Left Parietal WM	1.56 ± 0.10	1.56 ± 0.06	−0.33	77	0.746
Cerebellar vermis	0.94 ± 0.06	0.94 ± 0.06	−0.07	90	0.946
		*F* = 8.12,v1 = 5,v2 = 86,*P* < 0.001^*c*^			
Number of corrects	30.37 ± 3.73	37.78 ± 2.37	−11.58	82	<0.001^*b*^
Perseverative errors	11.49 ± 3.39	6.04 ± 2.44	8.87	92	<0.001^*b*^
Random errors	6.18 ± 2.64	4.18 ± 1.34	4.70	72	<0.001^*b*^
Categories completed	2.08 ± 1.59	4.93 ± 0.65	−11.52	65	<0.001^*b*^
		*F* = 37.74,v1 = 4,v2 = 89,*P* < 0.001^*d*^			
		*F* = 17.18,v1 = 9,v2 = 82,*P* < 0.001^*e*^			

^*a*^ Value is statistically significant (*P* < 0.05).

^*b*^ Value is statistically significant (*P* < 0.001).

^*c*^ Alcoholic group was different from controls for the variables of NAA/Cr levels.

^*d*^ Alcoholic group was different from controls for the variables of WCST scores.

^*e*^ Alcoholic group was different from controls for the variables including all the NAA/Cr levels and WCST scores.

### GRM3 SNP genotypes and NAA/Cr ratios

Because of unequal variances of certain genotypes for SNP rs6465084, we combined the individuals with the A/G heterozygous genotype and those with the G/G homozygous genotype into a G carrier group for analysis as described previously [13,14]. We performed a similar grouping for analysis of SNP rs1468412, in which the A/T heterozygotes and T/T homozygotes were combined into a T carrier group.

**Figure 3:**
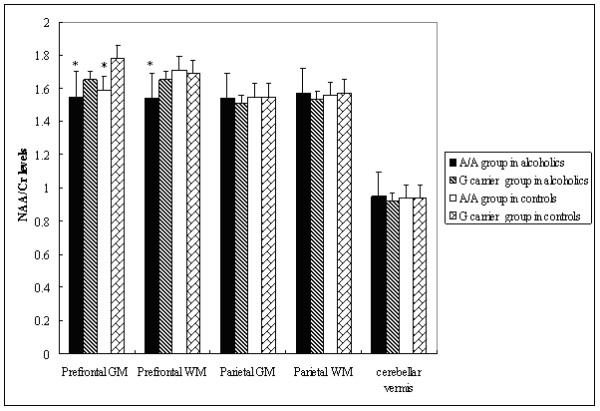
**SNP rs6465084 genotypes and NAA/Cr ratios.** NAA/Cr ratio levels in different brain regions of alcoholics and controls by SNP rs6465084 genotype. Statistically significant differences (P < 0.05) due to genotype are marked (*).

**Figure 4:**
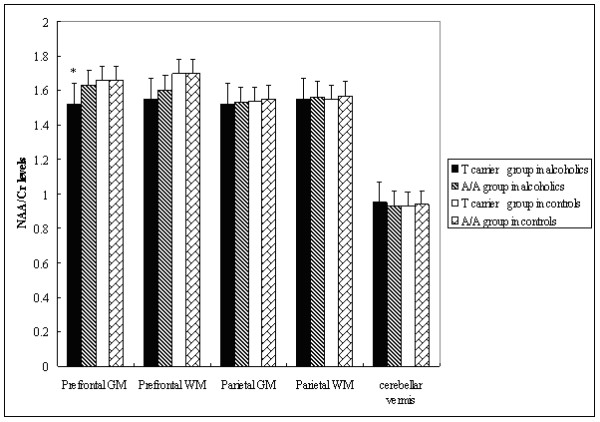
**SNP rs1468412 genotypes and NAA/Cr ratios.** NAA/Cr ratio levels in different brain regions of alcoholics and controls by SNP rs1468412 genotype. Statistically significant differences (P < 0.05) due to genotype are marked (*).

Among alcoholics, those having the A/A genotype for SNP rs6465084 were significantly different from the G carriers for the variables of NAA/Cr levels in the MANOVA (*F* = 5.26, v1 = 5, v2 = 41, *P* < 0.001). The A/A genotype group had statistically significantly lower NAA/Cr levels in the left prefrontal GM (*t* = −3.20, *df* = 41, *P* = 0.003) and in the prefrontal WM (*t* = −3.76, *df* = 47, *P* < 0.001) compared to the G carriers in unpaired *t* tests (Figure 3). No other NAA/Cr ratios in the other brain RoIs were significantly different between carriers of different rs6465084 SNP genotypes (Figure 3). For SNP rs1468412, alcoholics in the T carrier group weren’t different from those with the A/A genotype for the variables of NAA/Cr levels in the MANOVA (*F* = 1.65, v1 = 5, v2 = 41, *P* = 0.168). But we found that the T carrier group had lower NAA/Cr levels in the left prefrontal GM (*t* = −2.89, *df* = 47, *P* = 0.006) compared to those with the A/A genotype in unpaired *t* tests (Figure 4). No other NAA/Cr ratios in other brain RoIs were significantly different between carriers of different rs1468412 SNP genotypes. For SNP rs2299225, there were no significant differences in NAA/Cr levels in any brain RoIs between the T/T and G/T genotype groups in either the MANOVA or the unpaired *t* tests.

We performed the same analysis on the non-alcoholic control subjects and found that the group having the A/A genotype for SNP rs6465084 was significantly different from the G carriers for the variables of NAA/Cr levels in the MANOVA (*F* = 11.11,v1 = 5, v2 = 39, *P* < 0.001). The A/A genotype group for SNP rs6465084 had statistically significantly lower NAA/Cr levels in the left prefrontal GM (*t* = −7.42, *df* = 43, *P* < 0.001) compared to the G carrier group in unpaired *t* tests (Figure 3). No other NAA/Cr ratios in the other brain RoIs were significantly different between carriers of different rs6465084 SNP genotypes (Figure 3). For SNPs rs1468412 and rs2299225, there were no significant differences in NAA/Cr levels in any brain RoIs between different genotype groups in either the MANOVA or the unpaired *t* tests.

For all subjects, the A/A genotype group for SNP rs6465084 was significantly different from G carriers for the variables of NAA/Cr levels in the MANOVA (*F* = 9.11, v1 = 5, v2 = 86, *P* < 0.001). There were no significant differences between genotype groups for the variables of NAA/Cr levels for SNPs rs1468412 and rs2299225.

### GRM3 SNP genotypes and neurocognition

Among alcoholics, those having the A/A genotype for SNP rs6465084 differed significantly from the G carriers for the variables of WCST scores in the MANOVA (*F* = 31.35, v1 = 4, v2 = 44, *P* < 0.001). The A/A genotype of SNP rs6465084 was a predictor of poorer performance on EF skills tests in the areas of number of correct responses (*t* = −6.17, *df* = 47, *P* < 0.001), perseverative errors (*t* = 4.21, *df* = 47, *P* < 0.001), random errors (*t* = 2.88, *df* = 47, *P* = 0.006), and categories completed (*t* = −9.83, *df* = 47, *P* < 0.001) compared to G carriers (Table 4). For SNP rs1468412, alcoholics in the T carrier group did not differ from those with the A/A genotype for the variables of WCST scores in the MANOVA (*F* = 1.61, v1 = 4, v2 = 44, *P* = 0.188). Unpaired *t* tests indicated that the T carriers for SNP rs1468412 scored significantly lower in the number of correct responses (*t* = −2.20, *df* = 47, *P* = 0.033) and categories completed (*t* = −2.43, *df* = 47, *P* =0.019) than carrier of the A/A genotype (Table 4). There were no significant differences in WCST performance scores between the T/T and G/T genotype groups in an analysis of SNP rs2299225 using both MANOVA and unpaired *t* tests (Table 4).

**Table 4:** Scores achieved on the Wisconsin Card Sorting Test (WCST) by GRM3 SNP genotype

**WCST parameters**	**rs6465084**		**rs1468412**		**rs2299225**	
	**A/A group**	**G carrier group**	**T carrier group**	**A/A group**	**T/T group**	**G/T group**
**Alcoholic group**	(*n* = 31)	(*n* = 18)	(*n* = 19)	(*n* = 30)	(*n* = 37)	(*n* = 12)
Number of corrects	28.48 ± 3.16 ^*a*^	33.61 ± 2.03	28.95 ± 3.58 ^*b*^	31.27 ± 3.60	30.05 ± 4.05	31.33 ± 2.42
Perseverative errors	12.68 ± 3.54 ^*a*^	9.44 ± 1.82	12.53 ± 3.91	10.83 ± 2.90	11.59 ± 3.57	11.17 ± 2.89
Random errors	6.87 ± 2.87 ^*a*^	5.00 ± 1.68	6.58 ± 3.06	5.93 ± 2.36	6.41 ± 2.87	5.50 ± 1.68
Categories completed	1.10 ± 0.98 ^*a*^	3.78 ± 0.81	1.42 ± 1.35 ^*b*^	2.50 ± 1.61	2.00 ± 1.51	2.33 ± 1.87
	*F* = 31.35, v1 = 4, v2 = 44, *P* < 0.001 ^*c*^		*F* = 1.61, v1 = 4, v2 = 44, *P* =0.188		*F* = 0.47, v1 = 4, v2 = 44, *P* = 0.757	
**Control group**	(*n* = 29)	(*n* = 16)	(*n* = 18)	(*n* = 27)	(*n* = 30)	(*n* = 15)
Number of corrects	37.72 ± 2.46	37.88 ± 2.28	37.67 ± 2.43	37.85 ± 2.38	37.67 ± 2. 50	38.00 ± 2.17
Preservative errors	5.25 ± 1.88	6.48 ± 2.63	6.17 ± 2. 43	5.96 ± 2.49	6.30 ± 2.56	5.53 ± 2.17
Random errors	4.88 ± 1.20 ^*a*^	3.79 ± 1.26	4.17 ± 1.34	4.19 ± 1.36	4.03 ± 1.19	4.47 ± 1.60
Categories completed	5.00 ± 0.60	4.81 ± 0.75	4.78 ± 0.73	5.04 ± 0.59	4.93 ± 0.64	4.93 ± 0.70
	*F* = 3.28, v1 = 3, v2 = 41, *P* = 0.030 ^*d*^		*F* = 0.72, v1 = 3, v2 = 41, *P* =0.543		*F* = 0.51, v1 = 3, v2 = 41, *P* = 0.676	
	*F* = 3.43,v1 = 4,v2 = 89,*P* = 0.012 ^*e*^		*F* = 0.73,v1 = 4,v2 = 89,*P* = 0.574		*F* = 0.58,v1 = 4,v2 = 89,*P* = 0.681	

^*a*^ Value is statistically significantly different (*P* < 0.01) than that found in the comparison group for that SNP.

^*b*^ Value is statistically significantly lower (*P* < 0.05) than that found in the comparison group for that SNP.

^*c*^ The A/A genotype group for SNP rs6465084 was significantly different from the G carriers for the variables of WCST scores among alcoholics.

^*d*^ The A/A genotype group for SNP rs6465084 was significantly different from the G carriers for the variables of WCST scores among controls.

^*e*^ The A/A genotype group for SNP rs6465084 was significantly different from the G carriers for the variables of WCST scores in all the subjects.

Among non-alcoholic controls, the A/A genotype group for SNP rs6465084 was significantly different from the G carriers for WCST scores in the MANOVA (*F* = 3.28, v1 = 3, v2 = 41, *P* = 0.030). Those having the A/A genotype of SNP rs6465084 made statistically significantly more random errors (*t* = 2.79, *df* = 43, *P* = 0.008) than the G carriers in unpaired *t* tests. There were no other significant differences in WCST scores among the varying SNP genotypes among control subjects either in the MANOVA or in unpaired *t* tests (Table 4).

For all the subjects, the A/A genotype group for SNP rs6465084 was significantly different from the G carriers for the variables of WCST scores in the MANOVA (*F* = 3.43,v1 = 4,v2 = 89,*P* = 0.012). There were no significant differences between genotype groups for the variables of WCST scores for SNPs rs1468412 and rs2299225.

### The relationship between NAA/Cr levels and neurocognition

Among alcoholics, higher NAA/Cr levels in the prefrontal WM region were associated with a greater number of correct responses (*r* = 0.379, *P* = 0.007) and categories completed (*r* = 0.433, *P* = 0.002). Among controls, higher NAA/Cr levels in the prefrontal WM region were associated with a greater number of correct responses (*r* = 0.358, *P* = 0.015) and fewer perseverative errors (*r* = −0.431, *P* = 0.003). There were no other statistically significant relationships between NAA/Cr levels in other brain regions or in neurocognition.

After adjustment for the effect of genotypes, there were no significant relationships between NAA/Cr levels and neurocognition in the alcoholic subjects group. Among controls, higher NAA/Cr levels in the prefrontal WM region were associated with a greater number of correct responses (*r* = 0.365, *P* = 0.017) and fewer perseverative errors (*r* = −0.427, *P* = 0.004), excluding the influence of genotype.

## Discussion

In the present study, we assessed brain metabolite (NAA/Cr) levels and neurocognition performance in 49 non-smoking, active alcoholics and 45 non-smoking control subjects and analyzed the findings with respect to genotype at three GRM3 SNPs. The alcohol-dependent subjects in this study had lower NAA/Cr levels in left prefrontal GM and WM regions compared to controls and performed more poorly on EF skills tests than controls. Reduction of NAA levels in frontal WM has been demonstrated previously in active heavy drinkers [22]. Other studies in alcoholics abstinent for 3–40 days have shown lower NAA levels in the frontal lobes [36], thalamus [37], and cerebellum [33], suggesting neuronal injury, atrophied dendrites and/or axons, and/or mitochondrial dysfunction. Although the changes in brain NAA concentrations measured in these studies are small, the prefrontal and temporal regions appeared to be especially impacted by alcohol abuse. Meyerhoff et al. [22] demonstrated higher absolute concentrations of Cr in parietal GM of active heavy drinkers. Although we did not measure these, elevated absolute Cr levels might account for the lower NAA/Cr ratios observed in alcoholics in this study. The measurement of NAA/Cr ratios rather than absolute metabolite concentrations of NAA and Cr is a recognized limitation of this study. It has been shown that alcohol abuse is associated with a clear and consistent pattern of general cognitive deficits, which resolve to some extent with protracted abstinence [38]. The results of our study also suggest that brain damage in prefrontal regions and poor performance of EF tests were directly related to ethanol consumption. We did not measure blood alcohol levels of the participants at the time of MRS and cognitive testing since the influence of residual blood alcohol level on NAA and Cr levels is likely to be small [22]; however we do acknowledge that they could potentially affect cognitive test performance.

The effect of GRM3 SNPs on alcoholism has not been reported to our knowledge, therefore there are not specific studies regarding this. Our rationale for studying GRM3 in alcoholism is based on this SNP's involvement in other neuro-psychiatric diseases. The GRM3 SNPs in this study (rs6465084, rs1468412, and rs2299225) are the same as those reported to be associated with schizophrenia [11,13,15]. We observed a relationship between the A/A genotype of GRM3 SNP rs6465084 and lower NAA/Cr ratios in prefrontal GM and WM regions of alcoholics and in prefrontal GM of controls. These findings are consistent with prior studies in which this genotype was associated with lower NAA/Cr levels in the dorsolateral prefrontal cortex of healthy volunteers [14] and in which the A allele was associated with lower NAA/Cr levels in the prefrontal cortex of schizophrenia patients [13]. We observed a similar relationship between the T allele of SNP rs1468412 and reduced NAA/Cr levels in the prefrontal GM of alcoholics in this study. This finding has not been confirmed by others. The A/A genotype group for SNP rs6465084 was significantly different from the G carriers for all the variables of NAA/Cr levels in both alcoholics and controls, also indicating the obvious effect of A/A genotype for SNP rs6465084 on NAA/Cr levels. The glutamate signaling system has long been implicated in the acute and chronic effects of alcohol [6,7]. Based on evidence that GRM3 modulates synaptic glutamate, that it is a receptor for *N*-acetyl-aspartyl-glutamate, and that NAA is related to mitochondrial activity and to glutamate levels, the prefrontal reduction of NAA/Cr levels may reflect a genetically influenced alteration in glutamate neurotransmission [14].

While overall neurocognitive abilities were lower in alcoholics compared to controls, we also observed an effect of certain GRM3 SNP genotypes on cognitive abilities. In alcoholics, the A/A genotype of rs6465084 and the T allele of rs1468412 were associated with poorer EF. Among controls, the A/A genotype of SNP rs6465084 was associated with a single measured parameter of EF. Our findings agree with those of others, who have also suggested a relationship between SNP genotypes and individual variability in brain metabolite levels and inefficient prefrontal cortical functioning [39], and the evidence presented here of pattern of neurobiological and neurocognitive abnormalities [26,40,41]. Given that the A allele of SNP rs6465084 has been related to suboptimal glutamatergic signaling with an association between the A/A genotype of SNP rs6465084 and decreased prefrontal NAA/Cr levels and with poorer cognitive functioning in alcohol-dependent subjects, we believe that GRM3 gene variants may play a role in alcoholism.

In this study, we found that NAA/Cr levels in the prefrontal regions were associated with performance of EF in both alcoholic and control subjects. This is similar to previous findings in which lower NAA concentrations in the frontal lobe were associated with poorer performances on measures of EF skills and working memory in active-drinking and short-term abstinent alcoholics [22,26,33,36]. Lower test scores on WCST suggest poorer neurocognitive skills and functional brain damage in alcoholics, perhaps related to decreased NAA levels. Because we observed an obvious correlation between certain GRM3 SNP genotypes and NAA/Cr ratios and cognitive abilities, the relationship between NAA/Cr levels and neurocognition was also studied after the adjustment for the effect of genotype. The significant relationship between NAA/Cr levels and neurocognition among alcoholics disappeared after adjustment for genotype. Therefore the exact relationship between SNP genotypes, brain metabolite levels, and neurocognition is unclear, since these findings were not confirmed in the control group.

## Conclusions

We found that (1) non-smoking, active alcoholics without abstinence had lower NAA/Cr levels in prefrontal regions and performed more poorly on EF skills tests compared to control subjects; (2) certain GRM3 SNP genotypes were associated with lower levels of the glutamate metabolite NAA and with poorer performance on EF tests in alcoholics; and (3) NAA/Cr levels in prefrontal regions were associated with EF skills in both alcoholic and control subjects, but the significant relationship between NAA/Cr level and neurocognition among alcoholics disappeared after adjustment for the effect of genotype. Our results provide evidence that glutamate system dysfunction may play a role in the prefrontal functional abnormalities seen in alcohol dependence. It is possible that certain GRM3 SNP genotypes (i.e., the A/A genotype of rs6465084 and the T allele of rs1468412) may further lower NAA/Cr levels and EF skills in addition to the effect of alcohol. We hope to perform these studies on a larger population to verify these relationships.

## Abbreviations

Cr: Creatine; Cho: Choline-containing compounds; EF: Executive function; GM: Gray Matter; LDR: Ligase detection reactions; MRS: Magnetic resonance spectroscopy; MRSI: Magnetic resonance spectroscopic imaging; mGlus: Metabotropic glutamate receptors; GRM3: Metabotropic glutamate receptor 3; MANOVA: Multivariate analysis of variance; mI: myo-inositol; NAA: N-acetylaspartate; SNPs: Single nucleotide polymorphisms; WM: White Matter; WCST: Wisconsin Card Sorting Test.

## Competing interests

The authors declare that they have no competing interests.

## Authors' contributions

YX and DM contributed equally to this work. YX and DM designed the study and drafted the manuscript. YX and CT performed the experiment, analyzed the data and interpreted the results. ZW, LL, and FX participated in data collection. JH modified the manuscript and supervised the study. All authors read and approved the final manuscript.

## Supplementary Material

Additional file 1 Primer sequences for PCR-LDR of GRM3 region. Click here for file

Additional file 2 Probe sequences used in LDR. Click here for file
