# Supplementary material for: Effect of metabotropic glutamate receptor 3 genotype on N-acetylaspartate levels and neurocognition in non-smoking, active alcoholics
Source: Behav Brain Funct. 2012 Aug 21;8:42. doi: 10.1186/1744-9081-8-42 (PMC3508800; doi:10.1186/1744-9081-8-42)
Supplement: Additional file 1 — Primer sequences for PCR-LDR of GRM3 region. [file 1744-9081-8-42-S1.doc]

**Primer sequences for PCR-LDR of GRM3 region**

| SNP | Primer sequence (5’-3’) | PCR length |
| --- | --- | --- |
| rs6465084 | Forward: TGACACAAAGTTCTCTTTCCAA  Reverse: CTGCTAACCGCTGCTCTTTC | 104 |
| rs1468412 | Forward: TGATATGTTCCTTCAGCTTGC  Reverse: AGCCATGCAGTGGCAGAT | 201 |
| rs2299225 | Forward: TGCTTAGTATGTGCCTCTTATTGTG  Reverse: AGGCCCTAAGTGAGTTCATGT | 121 |
