# Supplementary material for: Effect of metabotropic glutamate receptor 3 genotype on N-acetylaspartate levels and neurocognition in non-smoking, active alcoholics
Source: Behav Brain Funct. 2012 Aug 21;8:42. doi: 10.1186/1744-9081-8-42 (PMC3508800; doi:10.1186/1744-9081-8-42)
Supplement: Additional file 2 — Probe sequences used in LDR. [file 1744-9081-8-42-S2.doc]

**Probe sequences used in LDR**

| SNP | Probe sequence (5’-3’) | LDR length |
| --- | --- | --- |
| rs6465084_modify | P-ATTTAATGGTAATTTGGAAAGAGAATTTTTTTTTTTTTTTTTT-FAM |  |
| rs6465084_A | TTTTTTTTTTTTTTTTTTATATCAATCCATGAAAAAGGCACAT | 86 |
| rs6465084_G | TTTTTTTTTTTTTTTTTTTTATATCAATCCATGAAAAAGGCACAC | 88 |
| rs1468412_modify | P-TACTGCCTATAACATTGCAAGCTGATTTTTTTTTTTTTTTTTTTT-FAM |  |
| rs1468412_A | TTTTTTTTTTTTTTTTTTTTATGCATTATGAGATAACAATCATTT | 90 |
| rs1468412_T | TTTTTTTTTTTTTTTTTTTTTTATGCATTATGAGATAACAATCATTA | 92 |
| rs2299225_modify | P-AGGTAGAGACCATGTTTTATTTATCTTTTTTTTTTTTTTTTTTTTTTTTTT-FAM |  |
| rs2299225_G | TTTTTTTTTTTTTTTTTTTTTTTTTTTCACATGTTGATTTAGTTGGCTTGC | 102 |
| rs2299225_T | TTTTTTTTTTTTTTTTTTTTTTTTTTTTTCACATGTTGATTTAGTTGGCTTGA | 104 |
